# Commentary: Rab GTPase: A New Mitotic Delivery Service

**DOI:** 10.3389/fcell.2015.00072

**Published:** 2015-11-17

**Authors:** Luisa Capalbo

**Affiliations:** Department of Pathology, University of CambridgeCambridge, UK

**Keywords:** Rab5, Rab11, mitosis, spindle envelope, membrane proteins

Mitosis is a crucial event that controls the equal partitioning of the genetic information between the two daughter cells. Failure in this process can cause various human genetic diseases and can contribute to cancer onset and development. For many years cell division studies focused on the identification and analysis of the mechanisms and signaling pathways that control microtubule dynamics and chromosome behavior, however in recent years other important cellular and regulatory processes have been implicated in various aspects of cell division and our traditional view of mitosis has changed. In particular several studies have highlighted the importance of membranes (Civelekoglu-Scholey et al., 2010; Poirier et al., 2010; Zheng, 2010; Schweizer et al., 2014) and membrane trafficking proteins (Royle et al., 2005; Liu and Zheng, 2009) during mitosis and revealed that many proteins known for their function in membrane trafficking in interphase have also a role during the mitotic process. In 2011 two papers reported a new role for the small GTPase Rab5 during mitosis and that this new function had been conserved through evolution (Capalbo et al., 2011; Serio et al., 2011). These papers found an unexpected new regulatory role for Rab5 GTPase in mitotic progression and regulation of membrane dynamics during nuclear envelope breakdown both in *Drosophila melanogaster* and human cells. They also reported that Rab5 is required for the correct alignment of chromosomes at the metaphase plate, an important step for the proper segregation of chromosomes.

Rab5 is one of the most studied small GTPases and is involved in numerous cellular processes, including membrane trafficking, signal transduction and cytoskeleton remodeling. It belongs to the most abundant subfamily of the small GTPases, the monomeric Ras-related proteins in brain (Rab) (Schwartz et al., 2007). Chavrier and colleagues first described Rab5 in 1990 (Chavrier et al., 1990). In this paper the authors described the localization of the yeast YPT1/SEC4 gene homolog in human cells and dubbed it Rab5. After this initial study several papers from the same group described Rab5 as an important molecule for different aspects of endocytosis, from vesicle docking to membrane fusion (Gorvel et al., 1991; Bucci et al., 1992; Zerial et al., 1992; Wandinger-Ness and Zerial, 2014). Recently Rab5 has also been found to be involved in autophagy and Alzheimer disease (Ravikumar et al., 2008; Ginsberg et al., 2010).

During mitosis, RNAi-mediated depletion of Rab5 induced chromosome alignment defects at the metaphase plate and delayed mitotic progression both in *Drosophila* and human cells. Rab5 positive vesicles accumulated around the spindle poles (Figure 1) and it was initially puzzling to reconcile this localization with the depletion phenotype. Earlier work in *Caenorhabditis elegans* identified a role of Rab5 in the re-organization of the nuclear envelope membranes just after nuclear envelope breakdown during the first embryonic division(s) (Audhya et al., 2007). Consistent with these findings, Rab5 depletion impaired nuclear envelope disassembly also in *Drosophila* and human cells. Lack of proper nuclear envelope breakdown delayed the release of important spindle proteins, ultimately affecting spindle function. In *Drosophila*, Rab5 associated *in vivo* with nuclear Lamin-B, which is not properly disassembled during nuclear envelope breakdown after Rab5 depletion. Furthermore, *Drosophila* Rab5 interacted with mushroom body defect (Mud), the *Drosophila* counterpart of the nuclear mitotic apparatus protein (NuMA) (Bowman et al., 2006). Mud/NuMA is an important microtubule-associated protein that accumulates at the spindle poles in prophase (Figure 1). Rab5 depletion caused a strong reduction or even absence of Mud at the spindle pole and consequently the spindle was not properly assembled. This, in turn, led to lack of kinetochore-microtubule tension that affected chromosomes alignment at the metaphase plate (Capalbo et al., 2011). Similar to *Drosophila*, human Rab5 was found to be required for chromosome alignment, but interacted with a different mitotic player, CENP-F required for chromosome congression (Liao et al., 1995). Depletion of all the human Rab5 isoforms (Rab5A, B, and C) reduced the accumulation of CENP-F at kinetochores, impairing the establishment of stable kinetochore-microtubule interactions (Serio et al., 2011).

**Figure 1:**
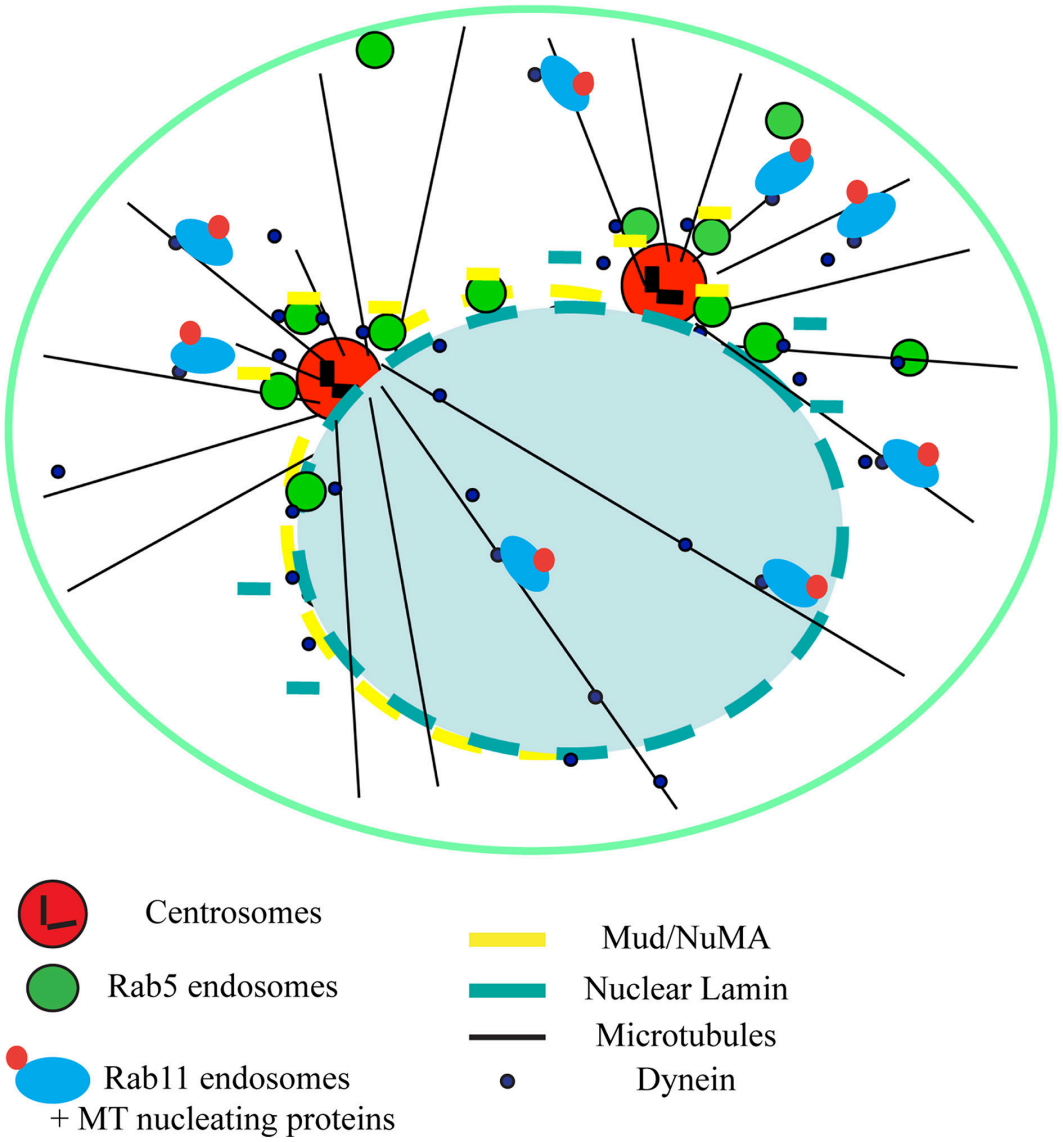
**Novel mitotic roles of Rab5 and Rab11 endosomes**. Schematic diagram illustrating the roles of Rab5 and Rab11 endosomes in binding and trafficking important mitotic proteins via dynein-mediated transport in prophase. In addition, is noteworthy that, during metaphase, Rab5 vesicles also move along spindle microtubules and transport CENP-F to kinetochores (not shown). See text for details.

Together, the findings of these papers indicated that incomplete disassemble of the nuclear envelope affected the release and localization of important proteins for spindle assembly and maintenance. They also showed that both *Drosophila* and human cells employ Rab5 proteins to direct membrane trafficking involved in the regulation and transport of key mitotic regulators important for the fidelity of chromosome segregation (Lanzetti, 2012; Figure 1). These works also suggested that Rab5 might function as part of a larger mechanism in which the interphase endocytic machinery is re-used for an alternative function during mitosis. Consistent with this hypothesis, a mitotic role was also recently found for another Rab family member, Rab11 (Hehnly and Doxsey, 2014). As already shown for Rab5 (Capalbo et al., 2011; Serio et al., 2011), depletion of Rab11 in human cells affected chromosome behavior. Rab11 depletion also changed mitotic spindle orientation, the plane of cell division and delayed mitotic progression. Furthermore, accumulation of important spindle pole proteins, such as major pericentriolar material proteins (γ-tubulin and pericentrin), was impaired (Hehnly and Doxsey, 2014). Finally, both Rab5 and Rab11 endosomes were found to carry MT nucleating/anchoring and regulatory proteins to the spindle poles through dynein-mediated transport (Capalbo et al., 2011; Serio et al., 2011; Hehnly and Doxsey, 2014; Figure 1).

In conclusion, these findings indicate that mitosis requires more than just microtubules and chromosomes, and increasing evidence over the last years has pointed out that a membranous system surrounding the mitotic spindle is important not only to confine important mitotic proteins around the spindle (Capalbo et al., 2011; Serio et al., 2011; Hehnly and Doxsey, 2014), but also as a mechanical support to hold the spindle structure in place (Poirier et al., 2010; Zheng, 2010; Schweizer et al., 2014). This spindle envelope is very important for mitosis as its elimination using laser microsurgery causes chromosome segregation errors consistent with defects in spindle assembly and kinetochore-microtubule attachments (Schweizer et al., 2015). Cells re-use during mitosis proteins known to be involved in different processes in interphase and the future challenge for the mitosis field is to identify these unexpected mitotic players.

## Conflict of interest statement

The author declares that the research was conducted in the absence of any commercial or financial relationships that could be construed as a potential conflict of interest.
